# Effect of sarcopenia combined with Metabolic Syndrome (MS) on the prognosis of intertrochanteric fractures: a retrospective clinical study

**DOI:** 10.1186/s12891-025-08931-2

**Published:** 2025-07-29

**Authors:** Cheng Zhang, Ding Wang, Xitao Wu, Qinrui Zhang, Lei Chen, Junzhe Lang, Congcong Wu

**Affiliations:** Department of Orthopedics, The First Affiliated Hospital of Wenzhou Medical University, Nanbaixiang, Ouhai District, Zhejiang Wenzhou, 325000 People’s Republic of China

**Keywords:** Metabolic syndrome, Sarcopenia, Intertrochanteric fractures, Prognosis

## Abstract

**Background:**

Intertrochanteric fractures pose significant health risks for elderly patients. While metabolic syndrome (MS) and sarcopenia independently impact postoperative outcomes, their synergistic effects on intertrochanteric fracture prognosis remain unclear. This study investigates the individual and combined influence of MS and sarcopenia on complications and functional recovery in elderly patients with intertrochanteric fractures.

**Methods:**

We retrospectively analyzed 460 elderly patients with intertrochanteric fractures, categorized into four groups: control (*n* = 174), MS (*n* = 122), sarcopenia (*n* = 89), and combined (*n* = 75). Preoperative—perioperative and postoperative data of the MS, sarcopenia, and combined groups were compared with the control group. Primary outcomes included 3-month Barthel Index (BI) and Harris Hip Score (HHS). Multivariate logistic regression identified predictors of poor recovery (HHS < 70).

**Results:**

The MS group had younger patients with higher body mass index (BMI), hypertension, diabetes prevalence, and longer surgery durations (*P* < 0.05). Sarcopenia and combined groups exhibited lower weight, BMI, handgrip strength (HS), appendicular skeletal muscle mass index (ASMI), and higher American society of Aneshesiologists (ASA) scores (*P* < 0.05). The MS, sarcopenia, and combined groups had higher incidences of pulmonary infections and pressure ulcers compared with the control group (*P* < 0.05). The sarcopenia and combined groups also had higher rates of organ failure (*P* < 0.05), with the combined group showing increased ICU admission (*P* = 0.003) and in-hospital mortality (*P* = 0.027). At three months post-discharge, the sarcopenia and combined groups exhibited higher mortality rates (*P* < 0.001). At the 3-month follow-up, the sarcopenia and combined groups had significantly lower HHS and BI compared to the control group (*P* < 0.05). Multivariate logistic regression identified sarcopenia alone (OR 6.50, 95% CI 2.56–20.10; *P* < 0.001) and combined with MS (OR 9.46, 95% CI 3.33–34.80; *P* < 0.001) as significant predictors of poor postoperative recovery.

**Conclusion:**

Sarcopenia significantly worsens postoperative prognosis in elderly intertrochanteric fracture patients, with synergistic deterioration when combined with MS. Chronic inflammation, insulin resistance, and muscle-bone metabolic dysregulation drive adverse outcomes. Comprehensive preoperative screening for sarcopenia and MS, coupled with tailored nutritional support and early rehabilitation, is critical to mitigate complications and improve functional recovery. These findings advocate for integrated care protocols targeting metabolic-musculoskeletal health in geriatric fracture management.

**Supplementary Information:**

The online version contains supplementary material available at 10.1186/s12891-025-08931-2.

## Introduction

Hip fractures, particularly intertrochanteric fractures, represent one of the most prevalent types of fractures among the elderly [1]. With increasing life expectancy and a growing global elderly population, the incidence of intertrochanteric fractures is projected to rise correspondingly [2]. These fractures not only pose an immediate health threat but also substantially compromise patients’ the long-term quality of life [3].

Metabolic syndrome (MS) and sarcopenia have emerged as critical global health concerns over recent decades. MS, characterized by central obesity, dyslipidemia, hyperglycemia, hypertension [4], exhibits a prevalence exceeding 30% among Chinese adults aged ≥ 60 years, closely tied to aging [5]. Its impact on postoperative outcomes in hip fracture patients remains debated. While MS is linked to heightened complication rates, its association with improved nutritional status—such as elevated albumin levels—may enhance postoperative recovery and reduce short-term mortality [6].

Sarcopenia, defined as a syndrome of progressive and generalized loss of skeletal muscle mass and strength largely due to aging [7], affects 1–29% of community-dwelling older adults and 14–33% of nursing home residents [8]. It is a well-established risk factor for perioperative complications, including pressure ulcers [9], pulmonary infections, and organ dysfunction [10], which extend hospital stays and elevate in-hospital mortality [11]. Moreover, sarcopenia leads to poorer functional recovery of the hip [12].

Despite extensive research on the individual effects of MS and sarcopenia on hip fracture patients, the specific impact of their coexistence on postoperative outcomes remains insufficient. Cross-sectional studies indicate a significant positive correlation between sarcopenia and MS [13]. MS is underpinned by insulin resistance and chronic inflammation, fueled by visceral adipose tissue accumulation. Skeletal muscle, responsible for 80% of postprandial glucose uptake, is highly insulin-sensitive; sarcopenia reduces muscle mass, impairs glucose utilization, and exacerbates insulin resistance with compensatory hyperinsulinemia [14].Visceral adipose tissue releases free fatty acids and proinflammatory cytokines tumor necrosis factor-α (TNF-α) and Interleukin-6 (IL-6), which disrupt phosphatidylinositol 3-kinase/protein kinase B (PI3K/Akt) signaling pathway and activate ubiquitin–proteasome-mediated muscle proteolysis [15]. Concurrently, TNF-α and IL-6 suppress mammalian target of rapamycin (mTOR) signaling, inhibiting muscle protein synthesis while upregulating atrophy-related genes [16]. Chronic inflammation further amplifies insulin resistance via c-Jun NH2-terminal kinase/nuclear factor kappa-B (JNK/NF-κB) pathway activation, promoting lipolysis and inflammatory cytokine secretion [17]. This interplay traps patients with coexisting MS and sarcopenia in a vicious cycle of inflammation and metabolic dysfunction [18].

Therefore, this study investigates the independent and synergistic effects of MS and sarcopenia on postoperative complications and functional recovery in elderly patients with intertrochanteric fractures, aiming to inform targeted clinical strategies for this vulnerable population.

## Methods

### Patients

This study enrolled 2048 elderly patients (aged ≥ 60 years) diagnosed with intertrochanteric fractures at the First Affiliated Hospital of Wenzhou Medical University, China, between January 2014 and December 2022.

#### Inclusion criteria comprises

1. American Society of Anesthesiologists (ASA) score ≤ 4.
2. Fresh intertrochanteric fractures (injury to admission time ≤ 14 days) treated with Proximal Femoral Nail Antirotation (PFNA).
3. Underwent dual-energy X-ray absorptiometry (DXA) for body composition analysis and handgrip strength measurement during hospitalization.
4. Complete follow-up records for at least six months post-discharge.

#### Exclusion criteria comprises

1. Multiple injuries.
2. Open fractures.
3. Severe pre-existing diseases and organ dysfunction.
4. Previous hip surgery.

(5) Pre-fracture hip mobility impairments.

**Fig. 1:**
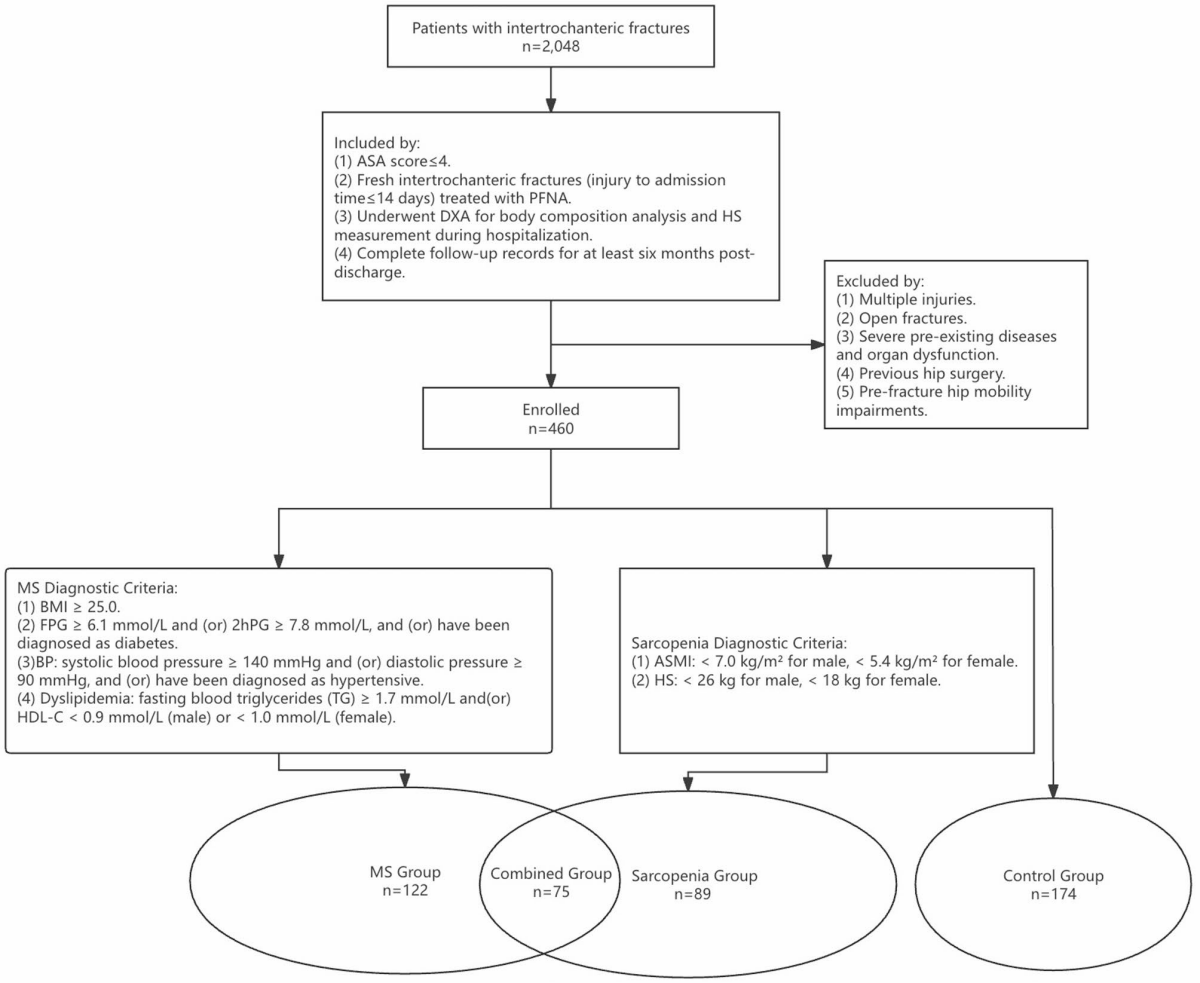
Study patient flow chart

Following these criteria, a final cohort of 460 patients was selected for analysis (Fig. 1). This research, including patient selection and data collection, was conducted after obtaining approval from the Wenzhou Medical University Hospital Institutional Review Board (KY2024-R151).

### Clinical data

#### Preoperative—perioperative data

Baseline characteristics were collected, including age, gender, length of hospital stay, hospitalization costs, medical history, smoking and alcohol history, injury to the time of surgery, surgery types, surgery duration, American Society of Anesthesiologists (ASA) score, anesthesia types, and relevant laboratory test indicators.

Surgery types comprise:

1. Open reduction.
2. Closed reduction.

Anesthesia types comprise:

1. General anesthesia.
2. Non-general anesthesia.
3. Combined anesthesia.

Laboratory indicators comprise:

1. Blood levels of vitamin D.
2. preoperative and postoperative counts of white blood cells, neutrophils, lymphocytes, hemoglobin, total protein, albumin, triglycerides, high-density lipoprotein cholesterol (HDL-C), and low-density lipoprotein (LDL).

Perioperative adverse events comprise:

1. Blood product usage, including red cell concentrates (RCCs), plasma, and albumin.
2. Perioperative pulmonary infection.
3. Venous thromboembolism (VTE) events.
4. Pressure ulcer.
5. Organ failure.
6. ICU admission.
7. Perioperative death.

#### Postoperative data

Primary outcomes were assessed three months post-discharge using the Barthel Index (BI) and Harris Hip Score (HHS).

The BI evaluates the patient's ability in 10 activities of daily living, with a total score of 100 indicating complete independence. The scores are categorized as follows: 0–20 (total dependence), 21–60 (dependence), 61–90 (moderate dependence), 91–99 (slight dependence), and 100 (complete independence) [19].

The HHS assesses hip function based on pain, functional capacity, absence of deformity, and range of motion [20]. Scores are classified as < 70 (poor), 70–79 (fair), 80–89 (good), and 90–100 (excellent) [21].

### Diagnostic criteria and grouping

MS was diagnosed using the China Diabetes Society (CDS) criteria [22], requiring three or more of the following:

1. Body mass index (BMI) ≥ 25.0 kg/m^2^.
2. Elevated serum glucose: fasting plasma glucose (FPG) ≥ 6.1 mmol/L and (or) 2-h plasma glucose (2hPG) ≥ 7.8 mmol/L, or prior diabetes diagnosis.
3. Blood pressure (BP): systolic BP ≥ 140 mmHg and (or) diastolic BP ≥ 90 mmHg, or prior hypertension diagnosis.
4. Dyslipidemia: fasting triglycerides (TG) ≥ 1.7 mmol/L and(or) high-density lipoprotein cholesterol (HDL-C) < 0.9 mmol/L (male) or < 1.0 mmol/L (female).

Sarcopenia was defined per the Asian Working Group for Sarcopenia (AWGS) criteria [23], requiring both:

1. Appendicular skeletal muscle mass index (ASMI) < 7.0 kg/m^2^ (males) and < 5.4 kg/m^2^ (females);
2. Handgrip strength (HS) < 26 kg (males) or < 18 kg (females).

Height, weight, appendicular skeletal muscle mass (ASM), and overall bone mineral density (BMD) were measured using a GE LUNAR DXA scanner (Model: Prodigy Primo, USA). ASMI was calculated as ASM divided by height squared (kg/m2) [24]. HS was measured on the dominant hand using an electronic handgrip dynamometer (EH101, Camry, Guangdong, China).

Patients were divided into four groups based on these criteria: (1) Control group (non-MS, non-sarcopenia). (2) MS group (MS only). (3)Sarcopenia group (sarcopenia only). (4) Combined group (MS and sarcopenia).

### Statistical analysis

Preoperative—perioperative and postoperative data of the MS, sarcopenia, and combined groups were compared with the control group. Statistical tests were selected based on data type and distribution.

Normally distributed quantitative data with homogeneous variance were expressed as mean ± standard deviation (x ± s) and analyzed using independent-sample t-tests. Non-normally distributed quantitative data were expressed as median (25th – 75th percentile) and analyzed using the Wilcoxon rank-sum test. Categorical data were presented as frequencies and percentages and analyzed with the chi-square test.

Patients were categorized into functional recovery (HHS ≥ 70) and incomplete recovery (HHS < 70) groups. Binary logistic regression models, adjusted for covariates, were used to evaluate the effects of MS, sarcopenia, and their coexistence on recovery outcomes, identifying independent predictors.

Statistical significance was set at *P* < 0.05.Analyses and visualizations were performed using R software (version 4.3.2) with the “gtsummary” and “forestploter” packages.

## Results

The study cohort consisted of 460 patients: 174 in the control group, 122 in the MS group, 89 in the sarcopenia group, and 75 in the combined group.

### Baseline characteristics

Baseline comparisons across groups are presented in Table 1.

**Table 1:** Patient demographic data by group

Variables	Control (*n* = 174)	MS (*n* = 122)	P^a^	Sarcopenia (*n* = 89)	P^b^	Combined (*n* = 75)	P^c^
Age (years)	81 (75, 85)	78 (73, 84)	0.011	84 (80, 89)	< 0.001	82 (77, 87)	0.4
Female (%)	98 (56%)	76 (62%)	0.3	40 (45%)	0.080	53 (71%)	0.034
length of hospital stay (days)	10.0 (8.0, 13.0)	10.0 (8.0, 12.8)	0.8	10.0 (8.0, 12.0)	0.8	10.0 (8.0, 12.5)	0.7
hospitalization costs (CNY)	30,859 (26,305, 36,362)	29,814 (26,311, 35,950)	0.5	30,916 (26,905, 35,159)	0.7	31,713 (25,975, 36,144)	> 0.9
Height (cm)	160 (155, 165)	160 (155, 165)	0.4	162 (155, 168)	0.11	158 (150, 165)	0.13
Weight (kg)	55 (50, 62)	60 (55, 65)	< 0.001	49 (44, 55)	< 0.001	50 (42, 55)	< 0.001
BMI (kg/m2)	21.6 ± 2.8	24.0 ± 3.4	< 0.001	18.82 ± 2.49	< 0.001	19.89 ± 3.50	< 0.001
HS (kg)	22.9 ± 6.0	23.3 ± 5.9	0.6	17 ± 5	< 0.001	15 ± 5	< 0.001
ASMI (kg/m2)	7.21 ± 1.15	7.37 ± 1.18	0.2	5.82 ± 0.83	< 0.001	5.40 ± 0.79	< 0.001
Hypertension (%)	85 (49%)	98 (80%)	< 0.001	43 (48%)	> 0.9	57 (76%)	< 0.001
Diabetes (%)	23 (13%)	73 (60%)	< 0.001	10 (11%)	0.6	30 (40%)	< 0.001
Smoking (%)	27 (16%)	12 (9.8%)	0.2	16 (18%)	0.6	6 (8.0%)	0.11
Drinking (%)	25 (14%)	18 (15%)	> 0.9	12 (13%)	0.8	4 (5.3%)	0.041
The injury-to-surgery interval (days)	4.00 (3.00, 6.00)	4.00 (3.00, 6.00)	0.8	4.00 (3.00, 6.00)	0.8	4.00 (3.00, 7.00)	0.8
Surgery duration (min)	59 (46, 86)	75 (56, 96)	< 0.001	66 (49, 82)	0.4	69 (55, 87)	0.066
ASA level ≥ 3 (%)	85 (49%)	65 (53%)	0.5	56 (63%)	0.03	47 (63%)	0.045
Anesthesia Type (%)			0.3		0.042		0.4
General	63 (36%)	41 (34%)		22 (25%)		28 (37%)	
Combined	24 (14%)	11 (9.0%)		8 (9.0%)		6 (8.0%)	
Non-General	87 (50%)	70 (57%)		59 (66%)		41 (55%)	
Surgery type (%)	41 (24%)	43 (35%)	0.028	24 (27%)	0.5	16 (21%)	0.7

*BMI* body mass index; *HS* Hand Grip Strength; *ASMI* Appendicular Skeletal Muscle Mass Index; *ASA level* American society of Aneshesiologists level; *MS* Metabolic syndrome

^a^Between Control and MS groups

^b^Between Control and Sarcopenia groups

^c^Between Control and Combined groups

Compared with the control group, the MS group was younger (*P* = 0.011), had higher weight and BMI (*P* < 0.001), a higher prevalence of hypertension and diabetes (*P* < 0.001), and longer surgery durations (*P* < 0.001). No significant differences were observed in sex distribution, length of hospital stay, hospitalization costs, handgrip strength (HS), appendicular skeletal muscle mass index (ASMI), smoking or alcohol use, time from injury to surgery, ASA classification, or anesthesia type (*P* > 0.05).

The sarcopenia group was older (*P* < 0.001), with lower body weight, BMI, HS, and ASMI (*P* < 0.001), higher ASA classifications (*P* = 0.030), and a higher proportion receiving non-general anesthesia (*P* = 0.042). No significant differences were found in length of hospital stay, hospitalization costs, prevalence of hypertension or diabetes, smoking or alcohol use, time from injury to surgery, or surgery duration (*P* > 0.05).

The combined group had a higher proportion of females (*P* = 0.034), lower body weight, BMI, HS, and ASMI (*P* < 0.001), a higher prevalence of hypertension and diabetes (*P* < 0.001), fewer alcohol users (*P* = 0.041), and higher ASA classifications (*P* = 0.045). No significant differences were observed in length of hospital stay, hospitalization costs, smoking history, time from injury to surgery, surgery duration, or anesthesia type (*P* > 0.05).

### Preoperative and postoperative laboratory parameters

Laboratory parameters for the MS, sarcopenia, and combined groups compared with the control group are presented in Table 2.

**Table 2:** Laboratory test data by group

Variables	Control (*n* = 174)	MS (n = 122)	P^a^	Sarcopenia (*n* = 89)	P^b^	Combined (*n* = 75)	P^c^
BMD (g/cm^3^)	0.67 (0.57, 0.77)	0.67 (0.57, 0.78)	0.7	0.65 (0.56, 0.74)	0.4	0.59 (0.52, 0.74)	0.019
VitD (ng/mL)	55 (44, 73)	52 (39, 61)	0.002	54 (41, 64)	0.049	51 (40, 66)	0.014
WBC (10^9/L)	8.28 (6.74, 10.28)	9.18 (7.33, 11.52)	0.007	8.83 (7.09, 10.40)	0.4	9.07 (7.52, 11.76)	0.013
NE (10^9/L)	6.32 (5.09, 8.12)	7.31 (5.34, 9.28)	0.012	7.12 (5.47, 8.47)	0.082	7.39 (5.75, 10.27)	0.002
LYM (10^9/L)	1.10 (0.85, 1.43)	1.16 (0.89, 1.47)	0.3	0.90 (0.69, 1.17)	< 0.001	1.03 (0.79, 1.33)	0.084
Hb (g/L)	107 ± 18	110 ± 19	0.089	105 ± 19	0.4	106 ± 19	> 0.9
Plt (10^9/L)	178 (145, 227)	199 (158, 262)	0.029	188 (152, 261)	0.3	194 (159, 233)	0.2
TP (g/L)	63.7 ± 6.2	64.9 ± 5.4	0.10	63 ± 7	0.5	64.3 ± 6.5	0.5
ALB (g/L)	34.7 (32.2, 37.0)	34.7 (32.7, 37.3)	0.4	33.9 (31.0, 36.6)	0.2	34.9 (31.7, 36.8)	> 0.9
TG (mmol/L)	1.12 (0.86, 1.47)	1.78 (1.19, 2.10)	< 0.001	0.95 (0.74, 1.31)	0.008	1.71 (1.12, 1.99)	< 0.001
HDL-C (mmol/L)	1.08 (0.87, 1.28)	0.89 (0.78, 1.05)	< 0.001	1.06 (0.87, 1.27)	> 0.9	0.94 (0.86, 1.12)	0.027

*BMD* Bone Mineral Density; *VitD* Vitamin D; *WBC* White Blood Cell; *NE* Neutrophils; *LYM* Lymphocytes; *Hb* Hemoglobin; *Plt* Platelets; *TP* total protein; *ALB* Albumin; *TG* Triglycerides; *HDL-C* High-Density Lipoprotein Cholesterol

^a^Between Control and MS groups

^b^Between Control and Sarcopenia groups

^c^Between Control and Combined groups

All three groups had lower preoperative vitamin D levels than the control group (*P* < 0.05). Only the combined group exhibited lower overall bone mineral density (BMD) (*P* = 0.019). The MS group had higher preoperative white blood cell (*P* = 0.007), neutrophil (*P* = 0.012), and platelet counts (*P* = 0.029). The sarcopenia group had lower lymphocyte counts (*P* < 0.001). The combined group showed higher white blood cell (*P* = 0.013) and neutrophil counts (*P* = 0.002).

### Perioperative adverse events

Perioperative adverse events are summarized in Table 3.

**Table 3:** Postoperative adverse events by group

Variables	Control (*n* = 174)	MS (*n* = 122)	P^a^	Sarcopenia (*n* = 89)	P^b^	Combined (*n* = 75)	P^c^
Transfused RCCs (%)	66 (38%)	38 (31%)	0.2	40 (45%)	0.3	36 (48%)	0.14
Transfused Plasma (%)	43 (25%)	16 (13%)	0.014	20 (22%)	0.7	20 (27%)	0.7
Transfused Albumin (%)	90 (52%)	55 (45%)	0.3	64 (72%)	0.002	53 (71%)	0.006
Wound infection	1 (0.6%)	3 (2.5%)	0.3	1 (1.1%)	> 0.9	2 (2.7%)	0.2
Pulmonary infection (%)	15 (8.6%)	20 (16%)	0.041	21 (24%)	0.001	20 (27%)	< 0.001
No. of VTE (%)	64 (37%)	50 (41%)	0.5	38 (43%)	0.4	36 (48%)	0.1
No. of pressure ulcer (%)	14 (8.0%)	19 (16%)	0.043	18 (15%)	0.031	16 (21%)	0.003
No. of organ failure (%)	7 (4.0%)	9 (7.4%)	0.2	10 (11%)	0.024	14 (19%)	< 0.001
No. of ICU admission (%)	7 (4.0%)	7 (5.7%)	0.5	8 (9.0%)	0.10	11 (15%)	0.003
No. of hospital death (%)	2 (1.1%)	2 (1.6%)	> 0.9	3 (3.4%)	0.3	5 (6.7%)	0.027
No. of death within 3 months (%)	4 (2.3%)	5 (4.1%)	0.5	21 (24%)	< 0.001	16 (21%)	< 0.001

*RCCs* red cell concentrates; *VTE* Venous thromboembolism

^a^Between Control and MS groups;

^b^Between Control and Sarcopenia groups

^c^Between Control and Combined groups

Regarding blood product usage, the MS group received less plasma than the control group, while the sarcopenia and combined groups required more albumin (*P* < 0.05). No significant differences in venous thromboembolism (VTE) incidence were observed across groups (*P* > 0.05). However, the MS, sarcopenia, and combined groups had higher incidences of pulmonary infections and pressure ulcers compared with the control group (*P* < 0.05). The sarcopenia and combined groups also had higher rates of organ failure (*P* < 0.05), with the combined group showing increased ICU admission (*P* = 0.003) and in-hospital mortality (*P* = 0.027). At three months post-discharge, the sarcopenia and combined groups exhibited higher mortality rates (*P* < 0.001).

### Comparison of postoperative recovery indicators

After excluding the patients died within 3 months, the postoperative recovery outcomes are shown in Tables 4.

**Table 4:** Postoperative follow-up index by Group

Variables	None (n = 162)	MS (*n* = 114)	P^a^	Sarcopenia (*n* = 63)	P^b^	MS**&**Sarcopenia (*n* = 55)	P^b^
HHS # 3 months	67 (59, 73)	65 (55, 72)	0.2	57 (50, 63)	< 0.001	57 (45, 64)	< 0.001
BI # 3 months	95 (85, 95)	90 (80, 95)	0.2	85 (75, 90)	< 0.001	80 (60, 85)	< 0.001

*HHS,* Harris Hip Score; *BI*, Barthel Index

^a^Between Control and MS groups

^b^Between Control and Sarcopenia groups

^c^Between Control and Combined groups

At the 3-month follow-up, the sarcopenia and combined groups had lower HHS and BI compared with the control group (*P* < 0.05). The MS group showed no significant differences in HHS or BI compared with the control group (P > 0.05).

### Association between MS, sarcopenia and postoperative functional recovery

**Fig. 2:**
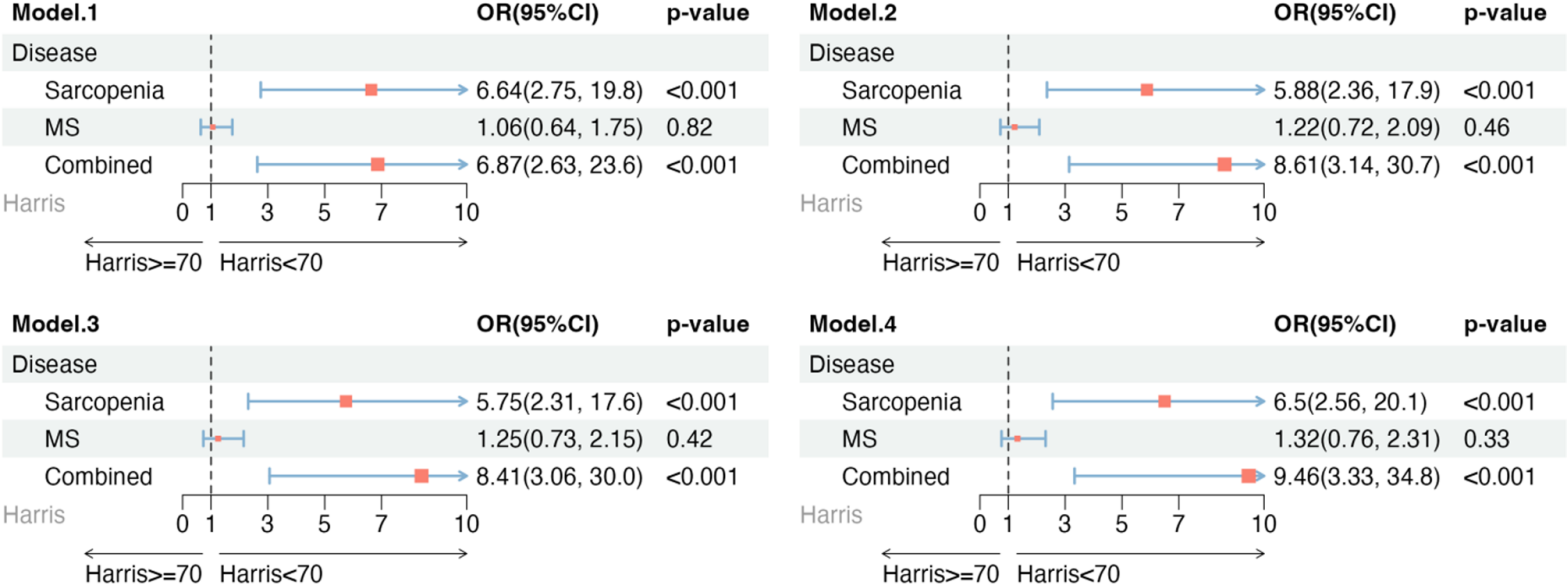
Forest Plot of Multivariate Logistic Regression. Model 1 was unadjusted. Model 2 was adjusted for *age_cate*, *sex.* Model 3 was adiusted for all variables in model 2 plus *smoking status*, *drinking status*. Model 4 was adjusted for all variables in model 3 plus *BMD*, *Pressure Ulcer*, *VTE*, *Wound infection*, *Pulmonary infection*

Multivariate logistic regression was used to identify independent risk factors for poor hip functional recovery (HHS < 70) in elderly patients with intertrochanteric fractures. Results are visualized in a forest plot (Fig. 2).

Four models were constructed: Model 1 included MS, sarcopenia, and their coexistence as primary exposure factors. Model 2 adjusted for age and sex. Model 3 further adjusted for alcohol consumption and smoking status. Model 4 included additional adjustments for BMD, pressure ulcers, VTE, and pulmonary infections. Sarcopenia alone and in combination with MS were significantly associated with an increased risk of poor postoperative functional recovery, with odds ratios (OR) of 6.50 (95% CI, 2.56–20.10; *P* < 0.001) and 9.46 (95% CI, 3.33–34.80; *P* < 0.001) in Model 4, respectively.

## Discussion

### Impact of metabolic syndrome on postoperative outcomes

MS is associated with increased incidence of pulmonary infections and pressure ulcers following intertrochanteric fractures in elderly patients. Our data revealed a higher incidence of pressure ulcers in the MS group (*P* = 0.043), possibly due to poor glycemic control, which impairs circulation and delays wound healing. Peripheral neuropathy linked to MS may reduce sensitivity to pressure and pain, limiting timely repositioning to alleviate pressure [25]. Microvascular complications in diabetic patients further lead to poor wound healing and infection resistance [26]. Additionally, MS patients exhibited increased rates of nosocomial infections, consistent with prior evidence identifying diabetes as a key risk factor for hospital-acquired infections in hip fracture cohorts [25]. Elevated blood glucose levels, a hallmark of diabetes, impair immune cell function, heightening susceptibility to postoperative pulmonary infections [27]. Moreover, MS patients required longer operative times (*P* < 0.001), potentially due to obesity, challenges in traction reduction, and a greater need for open reduction, which may contribute to higher pulmonary infection rates [28].

Despite elevated rates of pulmonary infections and pressure ulcers, isolated MS did not correlate with prolonged hospital stays, organ failure, ICU admissions, or mortality. This indicated that the complications usually appeared mild, and only need to be strengthened by repositioning and back-patting, nebulized sputum clearance, upgraded antibiotics, and pressure ulcer pads to obtain satisfactory therapeutic effect. Notably, MS patients demonstrated decent postoperative functional recovery, possibly due to younger age (P = 0.017) and greater muscle mass, enhancing physiological reserves and surgical tolerance.

### Impact of sarcopenia on postoperative outcomes

Sarcopenia is a well-documented predictor of poor postoperative outcomes [29]. In our study, sarcopenia patients exhibited higher rates of complications, including pressure ulcers (*P* = 0.031), pulmonary infections (*P* = 0.001), and organ dysfunction (P = 0.024), alongside increased albumin transfusions requirements (*P* = 0.002). Muscle atrophy in sarcopenia reduces subcutaneous fat padding, intensifying pressure on bony prominences and predisposing patients to skin breakdown. Concurrently, inadequate protein and micronutrient intake diminish tissue resilience and repair capacity [30]. Higher ASA scores and anesthesia type preferences underscored these patients’ poor surgical tolerance, compounded by advanced age and malnutrition, which exacerbated perioperative pulmonary infections and organ failure.

Sarcopenia is an independent risk factor for poor short-term hip function after surgery. Lower postoperative BI and HHS in sarcopenia patients indicate poorer early functional recovery, likely due to reduced muscle strength and coordination, hindering rehabilitation efforts [31]. Advanced age, postoperative complications, and limited functional exercise further contributed to elevated 3-month mortality in this group.

### Impact of combined MS and sarcopenia on postoperative outcomes

Both MS and sarcopenia constitute significant barriers to post-operative rehabilitation following hip replacement surgery. The pathophysiological core of MS resides in insulin resistance coupled with systemic metabolic dysregulation, while sarcopenia manifests as progressive loss of both muscle mass and functional strength, critically impairing rehabilitation potential [31, 32]. These two conditions share underlying pathomechanisms characterized by chronic low-grade inflammation and persistent oxidative stress. The resultant inflammatory milieu disrupts the balance between muscle protein synthesis and degradation pathways, ultimately leading to accelerated muscle atrophy. Rehabilitation fundamentally after hip fracture depends on coordinated activation patterns of the quadriceps femoris and gluteus medius muscle groups. Interestingly, patients with isolated MS often maintain compensatory skeletal muscle reserves sufficient for completing essential rehabilitation protocols. Adequate physiological reserve also allows the patient to recover as quickly as possible from the blow of trauma and surgery. Patients with coexisting MS and sarcopenia exhibited distinct characteristics compared to those with MS alone, including lower grip strength, BMI, and ASMI. This group was older, leaner, and more malnourished, lacking the physiological reserves seen in isolated MS patients and facing amplified metabolic dysregulation. Inflammaging—chronic inflammation affecting muscle metabolism, autophagy, and mitochondrial function—drives sarcopenia progression [33]. Elevated white blood cell (*P* = 0.013) and neutrophil counts (*P* = 0.002), alongside reduced lymphocyte counts (*P* = 0.084), suggest a state of malnutrition, metabolic dysfunction, and persistent inflammation. Lower bone mineral density (BMD; *P* = 0.019) indicates diminished muscle-mediated mechanical loading, impairing bone remodeling and exacerbating osteoporosis, which severely limits postoperative functional recovery and self-care capacity.

Vitamin D plays a pivotal role in calcium homeostasis and bone metabolism by enhancing intestinal calcium absorption, facilitating renal calcium reabsorption, and directly regulating osteoblast-osteoclast activity during bone remodeling [34]. Vitamin D deficiency inhibits osteoblast differentiation and releases inflammatory factors (TNF-α and IL-6), leading to impaired callus formation and accelerated osteolysis around internal fixation [35]. Our study revealed substantial vitamin D level reductions across all three groups compared to controls. Notably, only combined group demonstrated significant declines in overall bone mineral density (BMD), which significantly impaired postoperative fracture healing and hindered rehabilitation progress.

These factors culminated in increased adverse events, higher ICU admission rates, elevated perioperative mortality, and slower functional recovery in the combined group. Greater comorbidity burden further prolonged hospital stays, increased nosocomial infections, and fueled a vicious cycle of deterioration [36, 37]. The synergy of MS and sarcopenia markedly complicates postoperative prognosis, presenting unique management challenges.

### Study limitations

This single-center study, with a relatively modest sample size, may limit the generalizability of findings across diverse populations and regions. Focusing on early postoperative outcomes of intertrochanteric fractures, it lacks long-term follow-up to evaluate sarcopenia’s prolonged effects. Additionally, the study did not explore how different intertrochanteric fracture subtypes influence functional recovery, warranting further investigation.

## Conclusion

This study offers critical insights for clinical practice, revealing that sarcopenia markedly impairs postoperative prognosis in elderly patients with intertrochanteric fractures, with the effect most pronounced when combined with metabolic syndrome (MS). The synergy of sarcopenia and MS yields the poorest postoperative outcomes, highlighting the necessity of comprehensive preoperative evaluations and individualized postoperative management to optimize recovery in this high-risk population. Incorporating these findings into clinical care can improve outcomes for elderly hip fracture patients by reducing complications and enhancing overall prognosis.

## Supplementary Information

Supplementary Material 1.

## Data Availability

The datasets of study are available as supplementary material in Excel format. Due to reasons of patient privacy protection and ongoing further research using the database, the full dataset is not publicly available. However, it can be obtained from the corresponding author upon reasonable request.

## References

[1] Zhang Z, Qiu Y, Zhang Y, et al. Global trends in intertrochanteric hip fracture research from 2001 to 2020: a bibliometric and visualized study. Front Surg. 2021;8: 756614. 34778363 10.3389/fsurg.2021.756614 PMC8581155

[2] T J, Kwek E B K. Are Intertrochanteric Fractures Evolving? Trends in the Elderly Population over a 10-Year Period [J]. Clin Orthop Surg, 2022, 14(1): 13–20. 10.4055/cios20204 PMC8858907 35251536

[3] Curtis EM, Moon RJ, Harvey NC, et al. The impact of fragility fracture and approaches to osteoporosis risk assessment worldwide. Bone. 2017;104:29–38. 28119181 10.1016/j.bone.2017.01.024 PMC5420448

[4] Saklayen MG. The global epidemic of the metabolic syndrome. Curr Hypertens Rep. 2018;20(2):12. 29480368 10.1007/s11906-018-0812-z PMC5866840

[5] Li R, Li W, Lun Z, et al. Prevalence of metabolic syndrome in mainland China: a meta-analysis of published studies. BMC Public Health. 2016;16: 296. 27039079 10.1186/s12889-016-2870-y PMC4818385

[6] Cichos KH, Churchill JL, Phillips SG, et al. Metabolic syndrome and hip fracture: epidemiology and perioperative outcomes. Injury. 2018;49(11):2036–41. 30236796 10.1016/j.injury.2018.09.012

[7] Dionyssiotis Y. Sarcopenia in the elderly. Eur Endocrinol. 2019;15(1):13–4. 31244904 10.17925/EE.2019.15.1.13 PMC6587902

[8] Cruz-jentoft AJ, Landi F, Schneider SM, et al. Prevalence of and interventions for sarcopenia in ageing adults: a systematic review. Report of the International Sarcopenia Initiative (EWGSOP and IWGS). Age Ageing. 2014;43(6):748–59. 25241753 10.1093/ageing/afu115 PMC4204661

[9] Cha YH, Song SY, Park KS, et al. Relationship between pressure ulcer risk and sarcopenia in patients with hip fractures. J Wound Care. 2022;31(6):532–6. 35678788 10.12968/jowc.2022.31.6.532

[10] Chen X, Shen Y, Hou L, et al. Sarcopenia index based on serum creatinine and cystatin C predicts the risk of postoperative complications following hip fracture surgery in older adults. BMC Geriatr. 2021;21(1): 541. 34641805 10.1186/s12877-021-02522-1 PMC8507107

[11] Chang CD, Wu JS, Mhuircheartaigh JN, et al. Effect of sarcopenia on clinical and surgical outcome in elderly patients with proximal femur fractures [J]. Skeletal Radiol. 2018;47(6):771–7. 29247259 10.1007/s00256-017-2848-6

[12] Sanad HT, Hamza SA, Metwaly RG, et al. Sarcopenia and related functional outcomes following hip surgery among Egyptian geriatric patients with hip fracture. Cureus. 2023;15(8): e43166. 37692743 10.7759/cureus.43166 PMC10484563

[13] Ishii S, Tanaka T, Akishita M, et al. Metabolic syndrome, sarcopenia and role of sex and age: cross-sectional analysis of Kashiwa cohort study. PLoS One. 2014;9(11): e112718. 25405866 10.1371/journal.pone.0112718 PMC4236117

[14] Cheng JJ, Liang LJ, Lee CC. Associations of appendicular lean mass and abdominal adiposity with insulin resistance in older adults: a cross-sectional study. PLoS One. 2024;19(5): e0303874. 38753649 10.1371/journal.pone.0303874 PMC11098336

[15] Bian AL, Hu HY, Rong YD, et al. A study on relationship between elderly sarcopenia and inflammatory factors IL-6 and TNF-alpha [J]. Eur J Med Res. 2017;22(1):25. 28701179 10.1186/s40001-017-0266-9 PMC5508730

[16] Kaiser MS, Milan G, Ham DJ, et al. Dual roles of mTORC1-dependent activation of the ubiquitin-proteasome system in muscle proteostasis. Commun Biol. 2022;5(1): 1141. 36302954 10.1038/s42003-022-04097-y PMC9613904

[17] Nandipati KC, Subramanian S, AGRAWAL DK. Protein kinases: mechanisms and downstream targets in inflammation-mediated obesity and insulin resistance. Mol Cell Biochem. 2017;426(1–2):27–45. 27868170 10.1007/s11010-016-2878-8 PMC5291752

[18] Bernabeu-Wittel M, Gomez-Diaz R, Gonzalez-Molina A, et al. Oxidative stress, telomere shortening, and apoptosis associated to sarcopenia and frailty in patients with multimorbidity. J Clin Med. 2020. 10.3390/jcm9082669. 10.3390/jcm9082669 PMC7464426 32824789

[19] Wade DT, Collin C. The Barthel ADL index: a standard measure of physical disability? Int Disabil Stud. 1988;10(2):64–7. 3042746 10.3109/09638288809164105

[20] Nilsdotter A, Bremander A. Measures of hip function and symptoms: Harris Hip Score (HHS), Hip Disability and Osteoarthritis Outcome Score (HOOS), Oxford Hip Score (OHS), Lequesne Index of Severity for Osteoarthritis of the Hip (LISOH), and American Academy of Orthopedic Surgeons (AAOS) Hip and Knee Questionnaire. Arthritis Care Res Hoboken. 2011;63(Suppl 11):S200–7. 22588745 10.1002/acr.20549

[21] Harris W H. Traumatic arthritis of the hip after dislocation and acetabular fractures: treatment by mold arthroplasty. An end-result study using a new method of result evaluation. J Bone Joint Surg Am, 1969, 51(4): 737–55. 5783851

[22] Lan Y, Mai Z, Zhou S, et al. Prevalence of metabolic syndrome in China: an up-dated cross-sectional study. PLoS One. 2018;13(4): e0196012. 29668762 10.1371/journal.pone.0196012 PMC5906019

[23] Chen LK, Liu LK, Woo J, et al. Sarcopenia in Asia: consensus report of the Asian Working Group for Sarcopenia. J Am Med Dir Assoc. 2014;15(2):95–101. 24461239 10.1016/j.jamda.2013.11.025

[24] Cheng KY, Chow SK, Hung VW, et al. Diagnosis of sarcopenia by evaluating skeletal muscle mass by adjusted bioimpedance analysis validated with dual-energy X-ray absorptiometry. J Cachexia Sarcopenia Muscle. 2021;12(6):2163–73. 34609065 10.1002/jcsm.12825 PMC8718029

[25] Afzali Borojeny L, Albatineh AN, Hasanpour Dehkordi A, et al. The incidence of pressure ulcers and its associations in different wards of the hospital: a systematic review and meta-analysis. Int J Prev Med. 2020;11:171. 33312480 10.4103/ijpvm.IJPVM_182_19 PMC7716611

[26] Asadi K, Tehrany PM, Salari A, et al. Prevalence of surgical wound infection and related factors in patients after long bone surgery: a systematic review and meta-analysis. Int Wound J. 2023;20(10):4349–63. 37424390 10.1111/iwj.14300 PMC10681458

[27] Yuan Y, Tian W, Deng X, et al. Elderly patients with concurrent hip fracture and lower respiratory tract infection: the pathogens and prognosis over different bedridden periods. J Orthop Surg Res. 2021;16(1): 246. 33849586 10.1186/s13018-021-02399-1 PMC8042877

[28] Lv C, Chen S, Shi T, et al. Risk factors associated with postoperative pulmonary infection in elderly patients with hip fracture: a longitudinal study. Clin Nurs Res. 2022;31(8):1454–61. 36082422 10.1177/10547738221114713

[29] Yoo JI, Kim JT, Park CH, et al. Diagnosis and management of sarcopenia after hip fracture surgery: current concept review. Hip & Pelvis. 2022;34(1):1–9. 35355632 10.5371/hp.2022.34.1.1 PMC8931950

[30] Carretero Gomez J, Galeano Fernandez TF, Vidal rios AS, et al. Malnutrition and sarcopenia worsen short- and long-term outcomes in internal medicine inpatients. Postgrad Med J. 2023;99(1168):56–62. 36828395 10.1093/postmj/qgad006

[31] Xu BY, Yan S, Low LL, et al. Predictors of poor functional outcomes and mortality in patients with hip fracture: a systematic review. BMC Musculoskelet Disord. 2019;20(1):568. 31775693 10.1186/s12891-019-2950-0 PMC6882152

[32] Lim S. Journal of obesity & metabolic syndrome: a new international journal targeting the pathophysiology and treatment of obesity and metabolic syndrome. J Obes Metab Syndr. 2017;26(2):81–3. 31089499 10.7570/jomes.2017.26.2.81 PMC6484904

[33] Livshits G, Kalinkovich A. Inflammaging as a common ground for the development and maintenance of sarcopenia, obesity, cardiomyopathy and dysbiosis. Ageing Res Rev. 2019;56: 100980. 31726228 10.1016/j.arr.2019.100980

[34] Wacker M, Holick MF. Vitamin D - effects on skeletal and extraskeletal health and the need for supplementation. Nutrients. 2013;5(1):111–48. 23306192 10.3390/nu5010111 PMC3571641

[35] Mendes MM, Botelho PB, Ribeiro H. Vitamin D and musculoskeletal health: outstanding aspects to be considered in the light of current evidence. Endocr Connect. 2022. 10.1530/EC-21-0596. 10.1530/EC-21-0596 PMC9578072 36048470

[36] Huang J, Ge H, Zhu X, et al. Risk factors analysis and nomogram construction for postoperative pulmonary infection in elderly patients with hip fractures. Aging Clin Exp Res. 2023;35(9):1891–9. 37365389 10.1007/s40520-023-02480-1 PMC10460316

[37] Rambani R, Okafor B. Evaluation of factors delaying discharge in acute orthopedic wards: a prospective study. Eur J Trauma Emerg Surg. 2008;34(1):24–8. 26815487 10.1007/s00068-007-6184-8

